# Changeable camouflage: how well can flounder resemble the colour and spatial scale of substrates in their natural habitats?

**DOI:** 10.1098/rsos.160824

**Published:** 2017-03-08

**Authors:** Derya Akkaynak, Liese A. Siemann, Alexandra Barbosa, Lydia M. Mäthger

**Affiliations:** 1 Marine Biological Laboratory, Bell Center, Woods Hole, MA 02543, USA; 2 Department of Mechanical Engineering, Massachusetts Institute of Technology, Cambridge, MA 02139, USA; 3 Oceanography and Applied Ocean Science, Woods Hole Oceanographic Institution, Woods Hole, MA 02543, USA; 4 Coonamessett Farm Foundation, 277 Hatchville Road, East Falmouth, MA 02536, USA

**Keywords:** summer flounder, *Paralichthys dentatus*, windowpane flounder, *Scophthalmus aquosus*, natural substrates, crypsis

## Abstract

Flounder change colour and pattern for camouflage. We used a spectrometer to measure reflectance spectra and a digital camera to capture body patterns of two flounder species camouflaged on four natural backgrounds of different spatial scale (sand, small gravel, large gravel and rocks). We quantified the degree of spectral match between flounder and background relative to the situation of *perfect camouflage* in which flounder and background were assumed to have identical spectral distribution. Computations were carried out for three biologically relevant observers: monochromatic squid, dichromatic crab and trichromatic guitarfish. Our computations present a new approach to analysing datasets with multiple spectra that have large variance. Furthermore, to investigate the spatial match between flounder and background, images of flounder patterns were analysed using a custom program originally developed to study cuttlefish camouflage. Our results show that all flounder and background spectra fall within the same colour gamut and that, in terms of different observer visual systems, flounder matched most substrates in luminance and colour contrast. Flounder matched the spatial scales of all substrates except for rocks. We discuss findings in terms of flounder biology; furthermore, we discuss our methodology in light of hyperspectral technologies that combine high-resolution spectral and spatial imaging.

## 1. Introduction

Animal colour and body pattern changes are particularly well known in many animals, including lizards, amphibians, crabs, cephalopods and fish. The functions that these colour changes play in thermoregulation, intra- and interspecific communication, and camouflage are well documented [1–6]. In flatfish (e.g. turbots, soles and flounders, which are all members of the order Pleuronectiformes), colour and pattern changes are achieved by changing the relative visibility of chromatophores (melanophores, xanthophores, erythrophores, iridophores and leucophores). Melanophores, as well as xanthophores and erythrophores, are predominantly under neural control, so that the relative visibility of these darker chromatophores that overlay chromatophores of lighter shades determines the appearance of a flatfish [7–9].

Effective camouflage obviously increases an animal's success as a predator as well as its ability to hide from potential predators. Elegant studies conducted in the early 1900s show the advantage of colour adaptation in protection against predators [10,11]. The ability to change colour and pattern varies between flatfish species, but there is good agreement in the literature that its use is to provide camouflage on the benthic substrate [8,12–16]. Flatfish are generally passive bottom-dwelling animals that serve a role as predators as well as prey. Larger fish (including larger members of their own species) and cetaceans are known to include flatfish in their diet. By contrast, many flatfish species are themselves sit-and-wait predators that feed on a variety of fish, cephalopod and crustacean species [17–19].

The colour and pattern changes of a few flatfish species have been studied in detail since the earlier part of the twentieth century. Sumner [12] studied several species of flounder and turbot to establish the limits of their colour and pattern adaptation. He placed them on a variety of natural and artificial backgrounds and provided a detailed visual inspection of the distinct spots and blotches and colours that these flatfish showed on the presented backgrounds. Sumner also commented on the degree of colour and spatial frequency match of fish placed on backgrounds, but a detailed analysis was not possible with the methodology available at the time. Hewer [14] described the colour and pattern changes of several flounder and sole species and offered insight into the role of colour and pattern changes in camouflage. He provided detailed drawings of the various spots and blotches contributing to body patterns, and three distinct groups of patterns were described: (i) orange, black and white spots, (ii) pale spots, and (iii) normal area and dark patches. In a study on turbot, plaice and sole, Lanzing [8] showed that the various types of chromatophores are distributed in a permanent and fixed array, giving the fish a preset number of available patterns from which they can select the one that is most appropriate on a given background. Saidel [15] identified five distinct areas (morphological markers) in the winter flounder *Pseudopleuronectes americanus* and described the results of using a photomultiplier tube (PMT) to measure overall reflectance (at the waveband of maximal sensitivity of the PMT, which was unspecified) from the fish and the adjacent background. He showed that the average reflectance from the fish is affected by the average reflectance and contrast of the background. Most recently, Kelman *et al*. [16] studied European plaice (*Pleuronectes platessa*) and showed that an increase in substrate contrast and spatial frequency had a positive influence on the expression of spots and blotches on the fish.

There are varying reports in the literature regarding the time it takes for a flatfish to change its colour or pattern; and these times are likely to be species specific. The tropical eyed flounder *Bothus ocellatus* can match the spatial frequency of background patterns to a fine degree in a surprisingly short period of time (2–8 s) [20]. Yet the ability of flatfish to adaptively change colour and/or pattern appears to be more subdued in temperate/colder water species. Sumner [12] reported that it generally took several days to completely adapt a flatfish (various Mediterranean and northern Atlantic turbot species) to a new background until no further changes are seen, although he mentioned that body colour/pattern changes could be detected within seconds of placing the fish on a background. Mast [13] reported that, in flounder (*Paralichthys* and *Ancylopsetta* spp.), changes were accomplished within a matter of hours, while Kelman *et al*. [16] reported that plaice could change their pattern in 10–15 min. All authors added that there was individual variation between fish of the same species and that the level of adaptation and the time it took to accomplish a complete change depended on the flatfish species in question, the background that was tested and the previous experience the animal had with the substrate. Flatfish were able to adapt to a particular background much more quickly when they had repeatedly been exposed to it [12,13]. Flatfish also appear to adapt to natural substrates containing colours that are part of the fish's colour repertoire much more quickly than artificial substrates that are unnatural to the fish (e.g. artificially coloured substrates, checkerboards, etc.) [12,13,15]. Indeed, when settling on substrates, some flounder species actively choose substrates that are closest in colour and spatial frequency to the repertoire of changeable skin colours and patterns they have available [21].

Interestingly, the question of whether flatfish match backgrounds spectrally and spatially has not been addressed quantitatively, and our objective was to fill this gap in knowledge. Sumner [12] suggested that based on visual inspection, the flounder and turbot species he worked on (including *Rhomboidichthys; Rhobus; Lopsetta; Paralichthys*) often appeared to match the colours of the background. Tyrie *et al*. [21] reported similar observations for peacock flounder (*Bothus lunatus*). In our laboratory study, we used a hand-held spectrometer to quantify the degree of spectral match of two locally available species of flatfish (summer flounder *Paralichthys dentatus* and windowpane flounder *Scophthalmus aquosus*) on their natural backgrounds. In addition, we compared the spatial scale (granularity) of the splotches on the body of the flounder to that of the background substrates using methodology that was developed for quantifying patterns on bird eggs and cuttlefish [22–24] and modified to study peacock flounder patterns [21]. Since flounder are both a prey and a predatory animal, and a number of marine animals would benefit from detecting a camouflaged flounder, we quantified the degree to which these flounder spectrally matched substrates in the eyes of relevant mono-, di- and trichromatic observers. Furthermore, we provide a short discussion of the use of modern hyperspectral imaging (HSI) equipment for these types of studies, especially since both HSI and spectrometry have advantages and disadvantages that are important to consider when formulating research questions and designing experiments.

## 2. Material and methods

### 2.1. Flounder collection and maintenance

This study used three adult summer flounder *Paralichthys dentatus* (size: all approx. 40 cm) and three adult windowpane flounder *Scophthalmus aquosus* (size: all approx. 30 cm) that were collected by otter trawl off the coast of Woods Hole, MA in Vineyard and Nantucket Sounds. They were housed in a circular tank (310 cm diameter; 80 cm tall), filled with recirculating seawater (approx. 16°C) and maintained on a 12 L : 12 D  schedule. The fish were fed a mixed diet of live and frozen squid and fish. When regular feeding was observed, fish were considered acclimated to captivity and ready for use in trials.

### 2.2. Experimental design and data collection

A rectangular tank (width: 112 cm; length: 255 cm; height: 60 cm) was subdivided, using grey PVC sheets, into four compartments of equal size (112 cm × 63 cm) that were large enough to comfortably house one flounder for the duration of the experiment. The area where the experimental tank was housed was surrounded with black plastic to reduce disturbance to the animals. The bottoms of the experimental compartments were covered with a 5–10 cm layer of substrate. Flounder have distinct spots on their bodies, and these were approximately 1–1.5 cm in diameter for the *S. aquosus* and 3–4 cm in diameter for the *P. dentatus* used in the experiments*.* Thus, we chose substrates that were within the size ranges of these spots, as well as substrates that were larger than those spots. Four substrates, collected from local beaches, were used: *sand* (sand grains in millimetre range with some small pebbles < 3 cm), *small gravel* (approximate size range 3–7 cm, with some smaller sand grains in between), *large gravel* (approximate size range 5–10 cm) and *rocks* (approximate size range 10–15 cm). These substrates are typical of the natural habitats of the flounder used in this study.

Flounder were placed individually into each compartment and allowed to acclimate for 2 h before spectral measurements were taken. In our study, we did not notice any colour or pattern changes after approximately 30 min from the time we placed the animal on the substrate. Animals were only used once per day. Over the period of one month, all six animals were exposed to all four substrates. There was no specific order for placing fish on the different substrates. After the acclimation time, spectral measurements and photographs were taken. To minimize the impact of our presence and not cause the fish to move or change their body colours or patterns, we moved slowly around the tanks and moved probes and cameras gradually. Data collection took approximately 15 min. Most fish remained settled for the duration of the trial. Occasionally, a fish would move and resettle in a different part of the tank; however, colour and/or pattern changes during this short (usually less than 5 s) resettling period were not observed. To complete a trial, fish had to remain settled for the duration of data collection. If an animal moved during data collection, we abandoned that particular dataset and started again once the animal had resettled. We took spectral reflectance measurements of flounder and background using a fibre-optic spectrometer (USB 2000, Ocean Optics, Inc., Florida; effective range of measurement: 350–750 nm) connected to a PC laptop. A 400 μm diameter fibre was used. The fibre was hand-held by one experimenter at a distance of approximately 1 cm, perpendicular to the specimen (i.e. underwater), measuring a circular area of 5.0 mm diameter (see eqns 4–6 in [25]). Thus, we ensured that a sufficiently large area of skin was measured and a reliable spectral signal was obtained, since the flounder body pattern spot sizes had at least twice that diameter. A second experimenter operated the laptop during measurements. Regular white balancing was done using a diffuse reflectance standard (Ocean Optics, WS-1). The reflectance standard was placed adjacent to the area that was being measured, so that both reflectance standard and object of interest were in the same light field. Unlike in the experiments of Mäthger *et al*. [26], where the spectral similarity of cuttlefish and substrates was quantified in an outdoor setting with a natural light field, we were restricted to using indoor tanks because the fish in this experiment were too big for our outdoor set-up. The holding and experimental tanks were situated in an alcove surrounded by large windows, thus, illumination was from natural daylight, supplemented by one indoor fluorescent ceiling light. However, due to the windows, the usable waveband of our dataset was restricted to 400–700 nm. Dark and light reference spectra were taken frequently to ensure that we controlled for changes in illumination.

**Figure 1.**
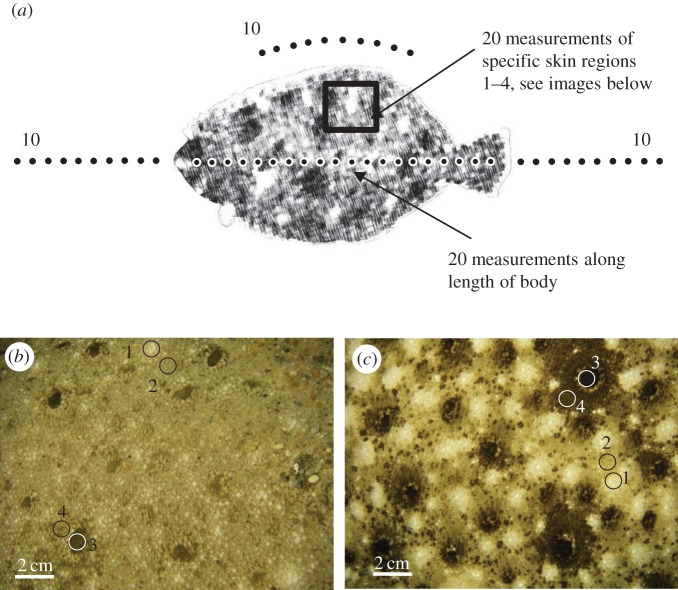
(*a*) Locations of spectral measurements. Ten measurements were taken of the substrate in a straight line in front of the head. Twenty measurements were taken along the length of the flounder, followed by 10 substrate measurements in a straight line behind the fin, as well as parallel to the dorsal fin. Twenty measurements of specific skin regions were also taken (see Material and methods section for detail). (*b*) Image of flounder body pattern typically shown on sand substrate. (*c*) Image of a flounder body pattern typically shown on gravel and rocks. We identified four distinct body areas that were expressed in a range of intensities depending on substrate: (1) white spot, (2) area surrounding white spot, (3) black spot and (4) area surrounding black spot (both images are *Paralichthys dentatus;* same four spot types were found in *Scophthalmus aquosus*).

**Figure 2.**
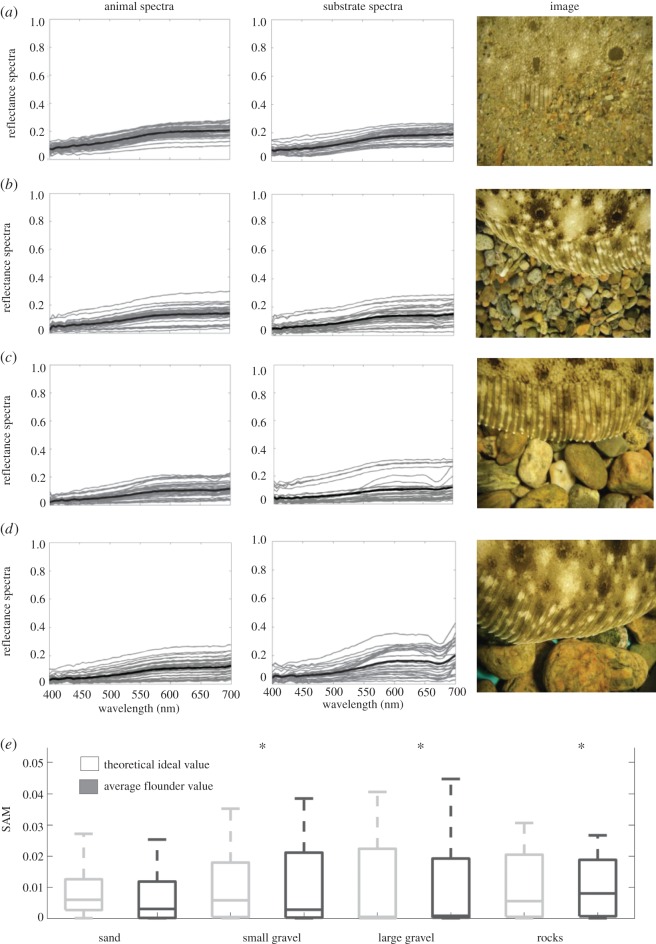
All individual spectral measurements of *P. dentatus* (left plots) and substrates (right plots), as well as an image showing a section of flounder and substrate. Grey lines are individual spectral measurements; heavy black line is the average. (*a*) Flounder on sand substrate, (*b*) flounder on small gravel substrate, (*c*) flounder on large gravel substrate, (*d*) flounder on rock substrate. (*e*) We computed the SAM metric for each substrate–flounder combination for ideal and actual scenarios and compared the similarity of these distributions (see Material and methods). Overall SAM values are low, indicating similar spectral shapes between the animal and the background spectra. Asterisk indicates that, using the Wilcoxon rank sum test at the 5% significance level, data come from distributions with equal medians.

**Figure 3.**
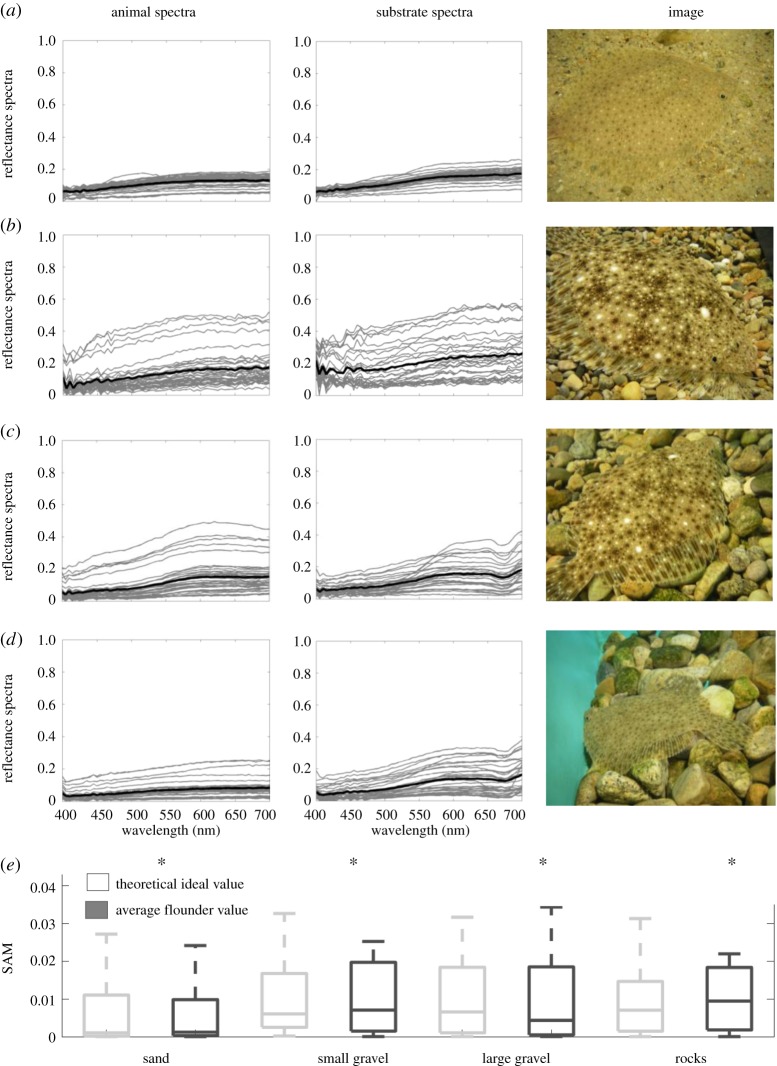
All individual spectral measurements of *S. aquosus* (left plots) and substrates (right plots), as well as an image showing a section of flounder and substrate. Grey lines are individual spectral measurements; heavy black line is the average. (*a*) Flounder on sand substrate, (*b*) flounder on small gravel substrate, (*c*) flounder on large gravel substrate, (*d*) flounder on rock substrate. (*e*) We computed the SAM metric for each substrate–flounder combination for ideal and actual scenarios and compared the similarity of these distributions (see Material and methods). Overall SAM values are low, indicating similar spectral shapes between the animal and the background spectra. Asterisk indicates that, using the Wilcoxon rank sum test at the 5% significance level, data come from distributions with equal medians.

We took spectral measurements of the fish as well as the adjacent substrate in the manner illustrated in figure 1*a*. We positioned our measurement points to capture as much of the variation as possible. (1) *Fish measurements*. Spectral measurements for each fish were taken in 20 locations along the anterior–posterior line, starting just behind the head, finishing just before the fin. To ensure we characterized the entire spectral signature of each flounder, we then took measurements of specific areas on the fish's body that are similar to the ‘morphological markers’ of Saidel [15]. These were often not located on the anterior–posterior line we routinely measured. Four such areas were identified: (i) white spot, (ii) area around white spot (or fish ‘background’), (iii) black spot, and (iv) area around black spot (figure 1*b*,*c*). Five measurements were taken of each area, for a total of 20 additional measurements. These areas could be shown by the fish with higher or lower intensity. For example, the white spots could vary between being large and conspicuous and being almost absent (i.e. taking on the colour of the surrounding body area). Similarly, the area around a black spot could be as black as the black spot itself; however, it could also take on the beige colour of the fish ‘background’ shade. We measured spectra from all areas, and grouped all of the fish spectra for a total of 40 measurements. (2) *Substrate measurements.* Ten spectral measurements were taken in a line in front of the head followed by 10 measurements behind the tail fin (figure 1*a*). We followed the anterior–posterior line of the fish when taking these substrate measurements. Additionally, we took 10 measurements of the substrate in a curve that was equidistant from the dorsal fin. In total, we took 30 measurements of the substrate. Figures 2 and 3 show all spectral data taken from one representative flounder on all four substrates for *Paralichthys dentatus* and *Scophthalmus aquosus*, respectively.

To document flounder patterning, we also took a series of photographs of the fish and adjacent background using a waterproof Pentax Optio W20. These photos were used for analysing the spatial scale of the fish body patterns and the background substrate.

### 2.3. Data analysis

#### 2.3.1. Chromaticity diagrams

To visualize the spectral match between flounder and their surroundings from the perspective of different visual systems, we used chromaticity diagrams for animals with colour vision following the methodology by Kelber *et al*. [27].

#### 2.3.2. Mathematical measure of spectral similarity

The Spectral Angle Mapper (SAM) metric, most commonly used in remote sensing, is a measure used to compare novel spectra to a specific known set of spectra; for example, to classify agricultural crops obtained via aerial surveying among a library of known crop spectra, e.g. corn versus soyabeans [28], or to classify coral reef organisms and substrates based on their known fluorescent signatures [29]. In this work, we used SAM for the quantification of similarity (or, dissimilarity) in two ways: first to compare the similarity of flounder and substrate *reflectance spectra*, and second for the comparison of *granularity spectra* of the flounder and that of the substrate (described below). Although SAM is purely a mathematical similarity metric, it has been shown to be moderately correlated to biological measures of colour similarity and can be used to obtain a rough estimate of how similar spectra are when spectral sensitivities of relevant observers are not known [30]. It is calculated as follows:

2.1 $$\text{SAM(}v_{1},v_{2}\text{)}=\cos^{-1}\left(\frac{v_{1}\cdot v_{2}}{∥v_{1}∥∥v_{2}∥}\right),$$

where *v*_1_ and *v*_2_ are the *N*-dimensional vectors, and ||·|| indicates their magnitudes, respectively [31]. It can be thought of as a measure of alignment between two *N*-dimensional vectors. This is easiest to visualize when *N* = 2; two vectors that are parallel (e.g. identical) have an angle, and therefore an SAM value of zero between them, while two that are perpendicular (most dissimilar) have an angle of *π*/2. The SAM metric is insensitive to differences in magnitude and only measures the similarity of spectral shape.

#### 2.3.3. Modelling observer visual systems

**Figure 4.**
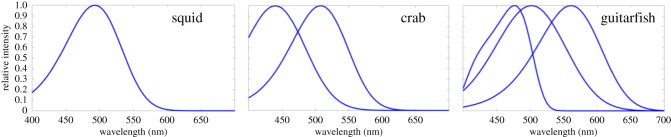
Spectral response curves of mono-, di- and trichromatic observers used in the study, normalized to have a value of 1 at peak wavelength.

Both *Paralichthys dentatus* and *Scophthalmus aquosus* are predatory fish but they also fall under the category of prey to other predators. Therefore, there are a number of observers that would benefit from being able to detect a camouflaged flounder. We chose three relevant species and, based on knowledge of their visual pigments, modelled how they might sense the colours of a camouflaged flounder, and whether they might be able to discriminate them against the substrate (figure 4): (i) squid *Doryteuthis pealeii,* (ii) green crab *Carcinus maenas*, and (iii) Atlantic guitarfish *Rhinobatos lentiginosus.* Squid have a single visual pigment at 493 nm [32], and they are a major dietary component of flounder. They also take great precautions to avoid being eaten once they have detected a flounder [17,18]. Green crabs have two visual pigments at 440 and 508 nm [33]. Depending on the life stages of the crabs and flounder, green crabs can be a predator of or prey for flounder. Atlantic guitarfish (a ray species from the Order Rajiformes, Suborder: Rhinobatiformes, Family: Rhinobatidae) most likely have three visual pigments at 477, 502 and 561 nm. This species is in the same family as *Rhinobatos* (*Glaucostegus*) *typus*, for which these three visual pigment absorbance maxima were reported [34]. Depending on the size of the flounder, these guitarfish could act as predators of flounder. Although there are relevant observers with more than three visual pigments (for example, the killifish *Fundulus heteroclitus—*a fish that flounder prey upon—has four visual pigments), we did not include any examples in our analysis because our spectral reflectance dataset was limited to 400–700 nm, and for the animals with more than three visual pigments, the fourth (etc.) pigment is generally found outside this 400–700 nm range. Previously, Akkaynak *et al*. [30] modelled photoreceptor ratios of 1 : 1 and 1 : 2 for dichromats and 1 : 1 : 1 and 1 : 2 : 2 for trichromats, which are typical fish retina cone mosaic patterns [35,36] and found that both scenarios yielded very similar results. Therefore, we limited our analysis to photoreceptor ratios of 1 : 1 for dichromats and 1 : 1 : 1 for trichromats.

#### 2.3.4. Modelling spectral discriminability

For chromatic contrast, relevant only for the di- and trichromatic observers (crab and guitarfish), we used the metric Δ*S* (with fraction 0.05) as defined by the Vorobyev–Osorio Receptor Noise Model [37]. Luminance contrast (Δ*L*) between the flounder body parts and background substrates, relevant for all three observers, was calculated as *L*_1_ − *L*_2_, where *L* = ln(*kQ*); *k* being the von-Kries adaptation coefficient and *Q* the quantum catch value. Spectra were collected under the same ambient conditions for each dataset, therefore the same von-Kries coefficient was applied to both luminance values (*k*_1_ = *k*_2_). Thus, $\Delta L=\ln k_{1}Q_{1}\text{/}k_{2}Q_{2}=\text{ln}\;Q_{1}\text{/}Q_{2}$. For Δ*L*, we only used the long wavelength receptor since it is considered to be responsible for luminance contrast in fish [38]. Quantum catch value was computed as follows:

2.2 $$Q_{i}=\int_{\lambda \min}^{\lambda \max}R(\lambda )I(\lambda )S_{i}(\lambda )\;\text{d}\lambda ,$$

where *R*(*λ*) is the reflectance spectrum of the feature of interest, *I*(*λ*) is the spectrum of the light source, *S_i_*(*λ*) is the spectral sensitivity of the *i*th photoreceptor (*i* = S,M,L), and *λ*_min_and *λ*_max_ are the lower and upper bounds of the visible spectrum, respectively. *S_i_*(*λ*) was based on the visual pigment template of Stavenga *et al*. [39] with the beta-peak shifted linearly, after Palacios *et al*. [40] using the equation given in Hart *et al*. [41].

Based on the Vorobyev–Osorio Receptor Noise Model, Δ*L* and Δ*S* values less than or equal to 1 (termed *just noticeable difference,* or *JND*) indicate that the spectra being compared are indistinguishable to the observer (see also [42,43]). However, this threshold is only meaningful when comparing one spectrum (i.e. one solid colour) to another. Yet, most natural habitats are composed of more than one colour, and most animal patterns contain more than one colour. In our study, we want to quantify the similarity of the entire spectral gamut the flounder can express to that of the substrate background. Under such conditions, how can we use the Vorobyev–Osorio Receptor Noise Model and evaluate and interpret discriminability between multiple colours? Previously, this kind of comparison has been done by comparing the percentage of all spectral combinations that result in a Δ*L* or Δ*S* value less than 1 (see fig. 3 and 4a in [30]). Here, we take a more structured approach by creating an ‘ideal’ camouflage scenario (described next) between the animal and the background, and comparing the resulting distributions of Δ*L* and Δ*S* to those between the ‘actual’ measurements of the animal and the background via a statistical test.

#### 2.3.5. Ideal camouflage scenario

What would perfect camouflage look like? Simplistically described, a flounder could be effectively concealed against a given background if it had the same spectral range and texture of the substrate (i.e. spatial patterning), assuming it did not cast any shadows. Here, we simplify this definition even further by omitting patterning and focusing only on perfect *chromatic* camouflage. We created a ‘perfect’ chromatic camouflage scenario by assuming that the fish and the background had the same spectral gamut. To obtain Δ*L* and Δ*S* distributions that we termed ‘ideal’, we combined all spectral measurements from each species of flounder taken on a given substrate, assumed that the background was composed of the same spectra, and calculated Δ*L* and Δ*S* for all possible pairwise combinations between them. Next, we calculated the same for the ‘actual’ spectral measurements for fish and substrate. Then we converted both the ideal and actual distributions to probability density functions. Finally, we used the Wilcoxon rank sum test [44] at the 5% significance level to test whether or not the ideal and actual scenarios came from distributions with equal medians, which gave us a mathematical tool to determine how good the flounders' camouflage was. For these comparisons, we identified two ways in which an actual distribution could be statistically equivalent to the ideal: (i) with the acceptance of the null hypothesis for the Wilcoxon rank sum test at the 5% significance level or (ii) if every Δ*L* or Δ*S* element in the *actual* distribution was less than 1, implying that all pairs of spectra were indistinguishable from each other. Before applying the Wilcoxon rank sum test, we normalized each bin in each distribution by sample size in that bin, obtaining the probability of members in that bin in the range 0–1. While more mathematical than biological, comparing the distribution of JNDs between the theoretical ideal scenario and the actual measured scenario allows us to gain more insights into the detectability of the flounder, as seen through the eyes of relevant observers, when multiple colours are involved. For our dataset, this approach yielded 1200 JND values per flounder per substrate (total of 40 fish spectra; 30 substrate spectra, resulting in 1200 spectral pairs); these are presented as box plots in figure 6 (luminance contrast) and figure 7 (chromatic contrast). Note that since the distributions were normalized for probability, the amplitudes shown no longer correspond to Δ*L* or Δ*S* thresholds.

#### 2.3.6. Quantification of spatial scale similarity

To characterize the patterns of the camouflaged flounder and their substrates, we used a Matlab granularity program developed originally for cuttlefish [22]. This granularity program has also been used to characterize bird egg patterns [24,45] and lizard throat signals [46]. Granularity analysis bins the pattern markings into seven spatial frequency bands, which range in size from large (Band 1) to very small (Band 7), and quantifies the contrast at each spatial scale. This provides a granularity spectrum or curve that summarizes the scale and contrast of light and dark elements in the pattern. The band-pass filtered images can be added together to reconstruct the original image, and examination of these filtered images provides a visual summary of the size of the pattern markings isolated in each band. Because the images did not always include a whole fish, the images were resized to a standard body length using prominent markings when the head and tail were not visible. All of the tank substrate in each image was used to analyse substrate patterns. We then used the *granularity spectrum* of each flounder and background substrate and represented them as a vector with seven values summarizing the relative contribution of pattern markings at each spatial scale. The SAM method (described previously) was used to calculate the spectral angle between each pair of animal versus substrate vectors. Lower SAM classifier values correspond to better matches between the animal and substrate granularity spectra (values range from 0 to *π*/2).

## 3. Results

### 3.1. Spectral similarity of flounder and substrates

**Figure 5.**
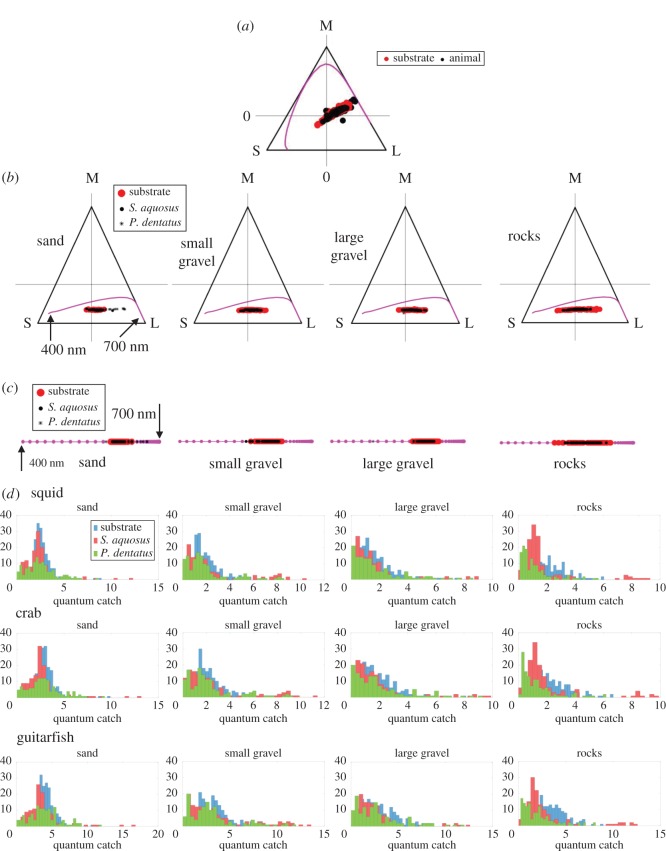
Chromaticity diagrams for animal vision. (*a*) Maxwell triangle drawn for the human visual system, showing the loci of flounder and substrate chromaticity coordinates obtained from radiance spectra. The magenta line shows the colour locus of monochromatic lights. Shown are all spectral data collected for both *P. dentatus* and *S. aquosus,* and all substrate spectra. (*b*) Maxwell triangles for trichromatic guitarfish. Even though the data measured from *P. dentatus* and *S. aquosus* are shown with different markers, they overlap significantly with each other and the background substrate. (*c*) Chromaticity diagram for the dichromatic crab. (*d*) Distribution of lightness for each visual system. Here, we used the quantum catch for the long wavelength receptor (following [47]) of each animal as proxy for lightness.

Flounder body pattern varied depending on substrate. On a given substrate, all individuals (three per species) showed the same spectral response. We identified four distinct body areas that, depending on the substrate, were expressed in a range of intensities: (i) white spot, (ii) area surrounding white spot, (iii) black spot, and (iv) area surrounding black spot (figure 1*b*,*c*). Overall, there was a good match between flounder spectral reflectance and the reflectance of all four substrates (sand, small gravel, large gravel and rocks), as can be seen when looking at the spectral plots as well as the image insets in figures 2 and 3 (which depict one representative dataset of each flounder species on all four substrates). For both *Paralichthys dentatus* (figure 2) and *Scophthalmus aquosus* (figure 3), the spectral dataset has a wide range (shown in both figures are spectra from all four body areas, ranging from nearly white to nearly black). In terms of wavelength, the spectra (both flounder and substrates) are broadband, with very few distinct spectral shades. Overall, most of the spectra are a general ‘brown’ shade (specifically, the spectra with low *y*-axis values). Some spectra with higher *y*-axis values are typical for light or white objects. In figures 2*c*,*d* and 3*c*,*d*, red rocks were clearly identified in the substrate spectral plots (see spectral peak in the range 600–650 nm). To further quantify spectral similarity between flounder and substrate spectra, we plotted the *xy*-coordinates of the tri-stimulus values of flounder body components and substrates on a Maxwell triangle (figure 5), which shows that all animal chromaticities fall within the gamut of those of the substrates, according to the human visual system. Figure 5*a* shows the equilateral triangle drawn for the spectral sensitivities representative of a human with normal colour vision and where the measured spectra fall on this diagram. Figure 5*b*,*c* shows the equivalent triangles for the trichromatic guitarfish and the dichromatic crab, respectively. In figure 5*d*, we show the distribution of lightness (the quantum catch of the longest wavelength receptor for each animal).

In addition, we used the SAM metric to quantify mathematical similarity between animal and substrate reflectance spectra for the ideal and actual camouflage scenarios for each experiment (figures 2*e* and 3*e*). Lower SAM values indicate higher spectral similarity, and across all experiments, animal spectra were most similar to those of sand.

### 3.2. Spectral similarity in the eyes of potential observers

**Figure 6.**
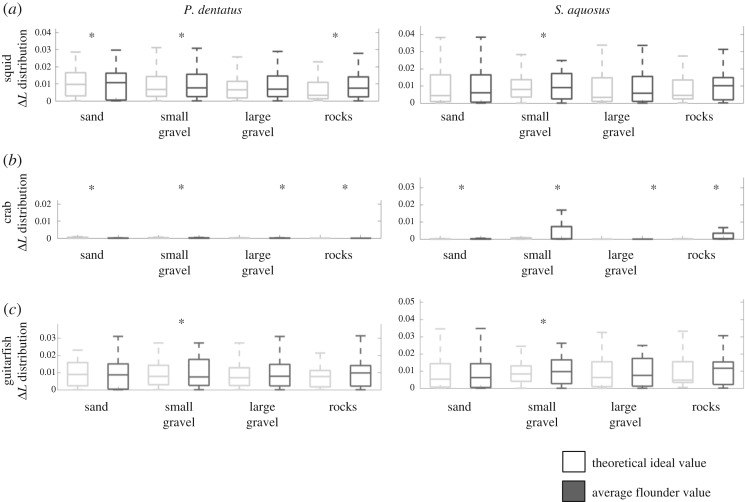
Box plots showing the actual and ideal Δ*L* distributions, calculated for three theoretical observers: monochromatic squid (*a*), dichromatic crab (*b*) and trichromatic ray (*c*). Results for each of these observers looking at *P. dentatus* are shown in the left panels; results for *S. aquosus* are shown in the right panels. The actual values for flounder, as determined by spectral measurements of flounder and substrates, are shown in dark grey; the theoretical ideal values (a calculation that considers the likelihood of detection if flounder were identical to background, i.e. a ‘perfect camouflage’ situation) are shown in light grey. Asterisks indicate whether the ideal and actual distributions are statistically equivalent.

**Figure 7.**
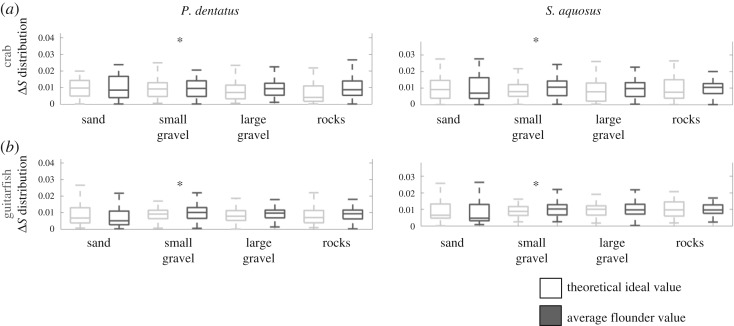
Box plots showing the actual and ideal Δ*S* distributions calculated for two theoretical observers: dichromatic crab (*a*) and trichromatic ray (*b*). Results for each of these observers looking at *P. dentatus* are shown in the left panels; results for *S. aquosus* are shown in the right panels. The actual values for flounder, as determined by spectral measurements of flounder and substrates, are shown in dark grey; the theoretical ideal values (a calculation that considers the likelihood of detection if flounder were identical to background, i.e. a ‘perfect camouflage’ situation) are shown in light grey. Asterisks indicate that the ideal and actual distributions are statistically equivalent.

In figures 6 and 7, the box plots show the distributions (for each species) for luminance (Δ*L*) and chromatic contrast (Δ*S*). In each figure, we show the actual distribution for flounder versus substrate spectra (dark grey boxes), and the theoretical ideal values (light grey boxes) that depict perfect camouflage. In each plot, the asterisks indicate that the ideal and actual distributions are statistically equivalent based on either of the two criteria we outlined in the Material and methods section. Note that since the distributions were normalized for probability, the amplitudes shown no longer correspond to Δ*L* or Δ*S* thresholds.

In terms of luminance contrast (figure 6), overall, the flounder matched the substrates well on most substrates. In figure 6, the most striking feature is that both actual flounder values and computed ideal values were very similar. The data were not all statistically equivalent, however (see point in Discussion). Notably, from the perspective of the crab visual system, our analysis showed that both species of flounder were indiscriminable on all substrates. Similarly, on small gravel, analysis showed that both species of flounder were indistinguishable from the background in the eyes of all three observers. The trichromatic guitarfish appeared to be able to detect the flounder on most substrates. However, when assessing luminance contrast, we only consider the long wavelength receptor, so the result for guitarfish luminance is likely due to the peak location of its long wavelength photoreceptor, rather than the presence of the other visual pigments (see also Discussion point about discriminability statistics).

With regard to chromatic contrast Δ*S* (figure 7), which concerns the two observers with colour vision (crab and guitarfish), we can see that both actual and ideal values are similar, again suggesting that the flounder are indiscriminable from their backgrounds. However, statistically, the distributions of the ideal Δ*S* values (depicting perfect camouflage) are not in the same range as the actual flounder values (although, visibly, they are very similar; see note in Discussion). The only exception is small gravel, on which the flounder appear to be indiscriminable from the background to crab and guitarfish.

### 3.3. Spatial match of flounder on substrates

**Figure 8.**
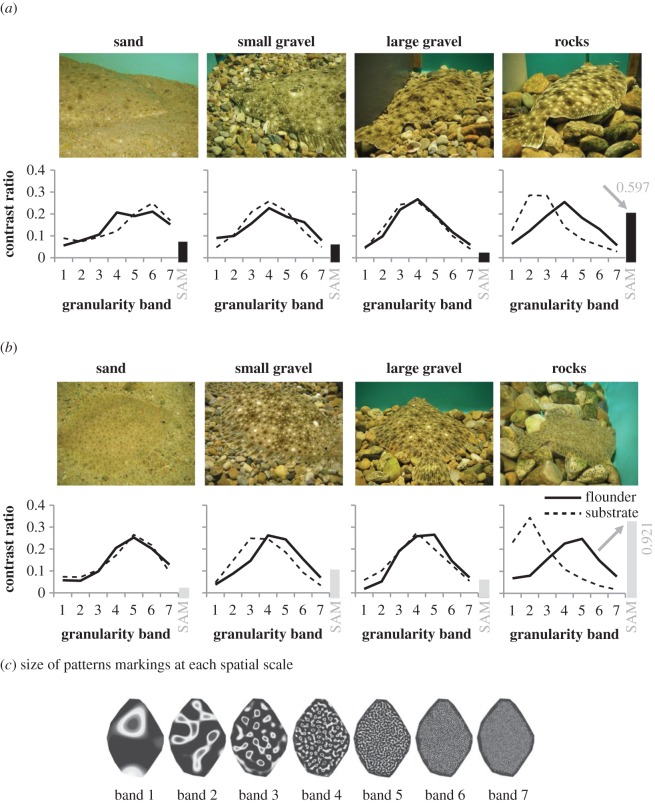
(*a*) Images of flounder and substrates (top) and granularity plots (bottom) of *P. dentatus.* (*b*) Same as (*a*) for S. *aquosus.* On sand, small gravel or large gravel, flounder matched the spatial scale of the substrate well. A clear mismatch can be seen for flounder on rocks. The bar plot on the right-hand side of the *x*-axis depicts the SAM value for each granularity band; see Material and methods for details of range. (*c*) For granularity analysis, flounder pattern markings are binned into seven spatial frequency bands, which range in size from large (Band 1) to very small (Band 7) [22].

When flounder settled on substrates with grain sizes similar to the sizes of their pattern markings (sand, small gravel or large gravel), they matched the spatial scale of the substrate well (figure 8). Bar plots in the far right side of the *x*-axis (figure 8) show the average SAM values for each flounder species on all four substrates. Examination of the granularity curves shows a clear mismatch between flounder and rock substrate curves, which is also confirmed by high SAM values of 0.921 for windowpane and 0.597 for summer flounder. The images in figure 8*a*,*b* also show a clear mismatch of both flounder species on rocks.

## 4. Discussion

Consideration of the effect of spectral signals in the eyes of potential observers has greatly advanced our understanding of visual communication and camouflage in animals [36,48–55]. In this study, we looked at the camouflage ability of summer flounder and windowpane flounder on four natural substrates (sand, small gravel, large gravel and rocks) in the eyes of biologically relevant observers (squid, crab and guitarfish, all of which can be predators or prey depending on the flounder life-history stage). While there have been a number of studies on flounder camouflage, the majority stem from the early to mid-1900s, with only a few recent studies [8,12–16,20]. To date, none have used a high-resolution optical instrument (e.g. a spectrometer) to investigate the degree of colour match flounder can achieve. To our eyes, flounder resemble substrates found in their natural habitats with high fidelity, and they are often not detectable in photographs. However, we are interested in assessing this resemblance through the eyes of biologically relevant observers that live in the same aquatic habitat, and which exert the evolutionary pressure that ultimately affects flounder camouflage. Flounder body patterning is complex in that it appears to achieve background resemblance in colour, intensity and pattern. How the relevant prey and predator animals may sense these features is likely to be different from how they appear to our visual system, and it is well known that using the human visual system to study animal patterning in nature often leads to highly misleading conclusions (e.g. [56]).

Our study adds a new approach to dealing with datasets for which multiple spectra need to be compared. In our dataset, only sand can be considered close to chromatically uniform---at least at the physical scale at which the substrates were measured; also, an approaching predator is unlikely to resolve sand grains from a distance. Gravel and rocks vary in size, shape and colour contrast, creating non-uniform, non-homogeneous backgrounds. This makes it a challenge to use the Vorobyev–Osorio Receptor Noise Model, and finding biologically meaningful interpretations of the Δ*L* and Δ*S* values, and in turn assessing detectability. Computing a theoretical baseline (a ‘perfect’ camouflage scenario) gave us a meaningful comparison of spectral gamuts. Our results show that all flounder and background spectra fall within the same gamut when assessed from a human's visual system, but this is not necessarily true for non-human observers. Whether or not a flounder can be chromatically discriminated from a given background not only depends on spectral similarity between the animal and the background, but also the peak and width of the spectral response curves of the observers, and our results certainly show that some animals are better at detecting particular background colours than others. We must point out that the data presented in figures 6 and 7 (actual fish versus computed ideal) appear to be strikingly similar, which would suggest that flounder were almost perfectly camouflaged to all observers. However, we only found statistical significance between actual fish measurements and ideal computed values (described as statistically equivalent) in a small number of pairs. For these pairs, confidence is high, so those truly have the same spectral distribution. The other pairs appear similar upon visual inspection but statistically they did not meet criteria to be equivalent. They may nevertheless be equivalent; the Wilcoxon rank sum test may not have been strong enough. Furthermore, while our data analysis may suggest that colour vision is more likely to help break flounder camouflage, we need to consider these statistical criteria before we can be confident that this is truly the case, and such a statement should certainly not be generalized.

It should be pointed out that our spectral dataset was taken at a distance of approximately 1 cm from the flounder/substrate and that it is extremely unlikely that any observer would be this close to a target. More likely, a predator would be many centimetres, even metres, away when swimming over, or moving by a flounder camouflaged on a substrate, thus visually taking in a much larger scene. Water type and turbidity also play a major role in underwater visibility. The natural habitats of these flounder are particularly turbid nearshore waters [57]. Therefore, it is conceivable that flounder might be even better camouflaged to potential observers in their natural habitats, where reduced visibility makes detection less likely.

In terms of spatial frequency, we found that flounder and substrate granularity bands are similar for sand, small gravel and large gravel. This result is similar to what has been reported for cuttlefish *Sepia officinalis*, where changeable body patterns are adjusted according to the spatial scale of the substrate [58]. This is the type of behaviour that is expected for animals that camouflage using the mechanism of background matching (versus disruptive coloration) [59–62]. On rocks, we found a clear mismatch, making flounder conspicuous on rocks (e.g. *S. aquosus* on rocks; figure 8*b*; right image and plot). It should be taken into consideration that flounder are benthic animals that often bury themselves when resting [63]. In our laboratory experiment, burying behaviour was only possible on the sand and small gravel substrate (although they did not bury themselves during experiments); the large gravel and rock substrates were too large to enable flounder to bury. To the best of our knowledge, it is unknown whether flounder actively choose substrates that allow them to bury themselves, or whether they equally well settle on substrates that do not permit burying. Certainly, some flounder species have been reported to choose substrates that better match their body pattern repertoire [21].

In our study, we investigated two components of camouflage: how similar animal and background are in terms of spectra and spatial frequency (granularity). We cannot assess the effectiveness of the overall camouflage—we quantified each of these components individually, and detected a good match; however, camouflage effectiveness is a function of the sum of several components including but not limited to colour and granularity. How spectral and granularity match, once combined, influence the effectiveness of camouflage in the eyes of a prospective observer remains an open question. Another interesting aspect is flounder size relative to camouflage ability. We used adult animals in our study but it would be valuable to compare our results across other life-history stages.

**Figure 9.**
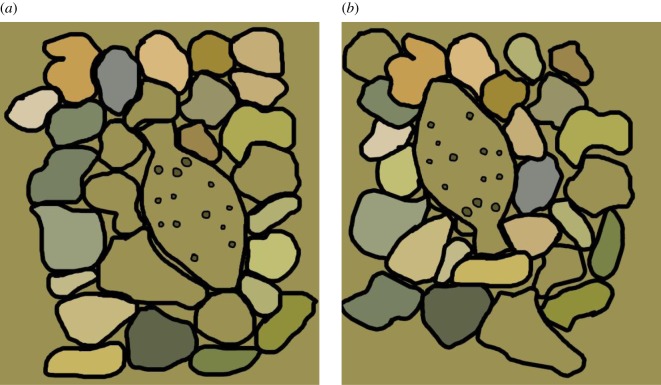
Two hypothetical, chromatically equivalent scenes of flounder settled on rocks. (*a*) Rocks that are the same colour as the flounder have been placed adjacent to it; (*b*) rocks are arranged so that their colours contrast with the colours of the flounder, making the flounder in (*b*) easier to detect even though the spectral gamut of both scenes (*a* and *b*) is identical.

Recently, several researchers have become interested in using HSI techniques to answer biological questions in living animals, in particular animal camouflage [47,64]. HSI is widely used in many industries and scientific fields that deal with stationary objects (e.g. [28,65,66]), but long exposure times and high costs still make their commonplace use in most research laboratories prohibitive. We did not have HSI available during the data collection that led to this paper. For our study, we used a spectrometer, which, to date, has been the most used instrument for quantifying coloration in animals, leading to many advances, particularly in camouflage (see table 1 in [25] for a partial list). However, without a carefully set-up grid for data acquisition and tedious data registration afterwards, spectrometers do not provide spatially coherent datasets, which are key to the evaluation of camouflage. To demonstrate the importance of spatial assessment in camouflage, in figure 9 we synthesized two chromatically equivalent scenes of flounder settled on rocks. In figure 9*a*, the rocks that are the same colour as the flounder have been placed adjacent to it, and in figure 9*b*, the rocks have been rearranged so that their colours are contrasting with the colours of the flounder. We argue that the flounder in figure 9*b* is easier to detect than the one in figure 9*a*, even though the spectral gamut in both scenes is identical. Thus, when a scene contains multiple colours, we often cannot reach conclusions about camouflage effectiveness simply by comparing point-by-point spectral measurements. This shortcoming can be remedied by the use of spatial imagers like a calibrated RGB camera or hyperspectral imager [67].

A comprehensive way of capturing an entire visual scene is using a hyperspectral imager since it records a given scene with high spectral and spatial resolution, without biasing it from the point of view of the human visual system the way off-the-shelf RGB cameras do [67]. A calibrated hyperspectral image can be filtered using the visual sensitivity curves of a non-human animal, obtaining that animal's view of the scene, from which the discriminability of the animal's body colours from the background colours in terms of JNDs can be extracted (e.g. [47,64]). HSI also has an advantage over a regular RGB-type camera in terms of pattern analysis, in that HSI captures information outside of the visible spectral range (note that some off-the-shelf cameras can be modified to extend their spectral sensitivity). Although RGB cameras still find use in characterizing biological specimens (e.g. [55]), extending the spectral sensitivity range beyond the human visible spectrum is biologically relevant (e.g. [68]). Thus, pattern information that would go unnoticed in an RGB image can be revealed using a hyperspectral imager. However, when interpreting how a pattern that is depicted in an RGB or hyperspectral image might be perceived by a non-human observer, one has to rely on models developed for human texture perception [69]; such models for animals do not exist, and doing psychophysics experiments with animals, especially fish, has been difficult [27,70]. Thus, while a hyperspectral imager offers an unbiased, high-resolution spectral photograph, and may reveal spatial bands that might not be detectable in RGB images, interpretation of body patterns, in the context of the background substrates, still relies on human vision-based techniques.

However, despite the obvious benefits of HSI, spectrometers still have advantages over HSI systems. In the last decade, commercially available spectrometers have continuously improved in terms of spectral bandwidth, sensitivity and speed. In terms of data quality, these spectra are generally of higher resolution than most hyperspectral imagers. The Ocean Optics USB 2000 spectrometer used in this study sampled the visible spectrum in 0.38 nm intervals, resulting in 790 measurements between 400 and 700 nm. By contrast, a commercially available hyperspectral imager, SpectraCAM, takes only eight measurements between 300 and 900 nm. A Surface Optics Corporation custom-built imager has 16 channels, with a spectral range from 360 to 660 nm, measuring at an interval of approximately 20 nm (R. Hanlon, MBL). In comparison, the hyperspectral imager used by Chiao *et al*. [47] samples the spectrum in 412 bins, but this is still half the resolution of a spectrometer.

The caveat, of course, is that spatial information is not instantly available when using a spectrometer. In theory, reconstructing a scene spatially from point-by-point spectral measurements is not impossible; however, it would require multiple optical fibres and spectrometers, so that data can be taken in a synchronized fashion. This is tedious and error-prone and obviously easier with HSI equipment. However, HSI is still in the early stages of development with regard to biological application, for which speed is often a critical component of data collection because of a target's mobility or colour-changing properties. Currently, a typical HSI scan takes of the order of several seconds to minutes to complete, which is acceptable for stationary targets (thus, HSI finds much use in medical and food industries) but more difficult for living biological specimens. Lastly, HSI equipment is still expensive and out of the range for many researchers. Thus, our study demonstrates an inexpensive way of fully characterizing two aspects of a camouflaged target in a complex environment; yet, our analysis can be taken further with a hyperspectral imager, and a more comprehensive and integrated evaluation of camouflage can be performed.

## Data accessibility

Data are available from the Dryad Digital Repository: http://dx.doi.org/10.5061/dryad.f51f5 [71].
